# Quantifying the Potential Impact of Food Fortification on Iron Intake, Hemoglobin Concentration and Anemia Prevalence Among Women of Reproductive Age in India

**DOI:** 10.1111/mcn.70181

**Published:** 2026-08-24

**Authors:** Hanqi Luo, Elizabeth Lambert, Xinrui Hu, Manpreet Chadha, Daniel López de Romaña, Mandana Arabi, Helena Pachón

**Affiliations:** ^1^ Hubert Department of Global Health, Rollins School of Public Health Emory University Atlanta USA; ^2^ Department of Epidemiology, Rollins School of Public Health Emory University Atlanta USA; ^3^ Department of Biostatistics and Bioinformatics, Rollins School of Public Health Emory University Atlanta USA; ^4^ UNICEF Brussels Belgium; ^5^ Nutrition International Ottawa Canada; ^6^ Food Fortification Initiative Atlanta USA

**Keywords:** anemia, fortification, iron, modeling, South Asia

## Abstract

Anemia is a public health burden, particularly in women of reproductive age (WRA). Food fortification, by adding micronutrients to foods, may alleviate anemia caused by nutritional deficiencies. To model the impact of iron‐fortified rice, wheat flour, and salt in India, we simulated three scenarios: no fortification, intermediate fortification (50% coverage for rice and wheat flour and 26.6% coverage for salt, with 100% industry compliance), and maximum fortification (100% coverage and compliance) in WRA. We first used a single‐day, 24‐h dietary recall survey from the National Nutrition Monitoring Bureau to simulate mean iron intake under these scenarios (*n* = 11,625). We then used data from the National Family Health Survey 4 (*n* = 667,258) to simulate hemoglobin changes due to increased iron intake, based on established parameters from a meta‐regression analysis. Subsequently, we calculated anemia prevalence. Compared to the no‐fortification scenario, the intermediate and maximum fortification scenarios were predicted to increase daily iron intake by 4.3–6.5 mg and 8.6–13.1 mg (35%–53% and 70%–106% increases), respectively, and decrease anemia prevalence from 52.6% to 45‐48% and 36‐43%, respectively. Our results suggest that combined fortification of rice, wheat flour, and salt with iron at varying levels of coverage and 100% compliance can improve iron intakes and hemoglobin concentration and thus reduce anemia prevalence among WRA in India.

## Summary

- This study is the first to model iron intake by women of reproductive age using 24‐h dietary recalls, rather than household food consumption surveys, to assess food fortification impact on hemoglobin concentration and anemia prevalence using the Indian government's current fortification standards.
- Iron fortified rice, wheat flour, and salt are predicted to increase hemoglobin concentration and subsequently reduce anemia prevalence, suggesting that iron fortification of rice, wheat flour, and salt can be an effective strategy to combat anemia in India.
- Even under an intermediate fortification scenario with partial coverage (50% for rice and wheat flour, 26.6% for salt), anemia prevalence among women of reproductive age is predicted to decrease from 52.6% to 45.1–47.6%, supporting the feasibility of meaningful public health impact without requiring full market coverage.

**Abbreviations**
CI: confidence interval
CNNS: Comprehensive National Nutrition Survey
DHS: Demographic and Health Surveys
FSSAI: Food Safety and Standards Authority of India
GDD: Global Dietary Database
ICDS: Integrated Child Development Scheme
NFHS‐4: National Family Health Survey ‐ 4
NNMB: National Nutrition Monitoring Bureau
OR: odds ratio
PDS: Public Distribution System
PM‐POSHAN: Pradhan Mantri Poshan Shakti Nirman (previously known as the Mid‐Day Meal/MDM program)
PP: percentage points
RDA: Recommended Dietary Allowance
SAS: Statistical Analysis System
SSNPs: Social Safety Net Programs
STROBE: Strengthening the Reporting of Observational Studies in Epidemiology
WRA: women of reproductive age

## 1 Introduction

Globally, an estimated 30% of women of reproductive age (WRA) suffer from anemia (Stevens et al. 2022). Anemia is characterized by insufficient hemoglobin in the body, which impairs the body's ability to transport oxygen (Gardner and Kassebaum 2020). Hemoglobin synthesis is a complex process that depends on several key nutrients, including copper, folate, iron, riboflavin, thiamine, vitamins A, B12, B6, C, E, and zinc (Badham et al. 2007). Nutritional anemia, the most common type of anemia, occurs when deficiency of one or more of these nutrients leads to hemoglobin insufficiencies (Gardner et al. 2023). Globally, approximately 20% of maternal mortality can be attributed to maternal short stature and iron deficiency (Gardner et al. 2023).

Nutritional anemia is particularly prevalent in South and Southeast Asia (Gardner et al. 2023; Sunuwar et al. 2020). A recent study estimated that 53.1% of women in India have anemia (Sunuwar et al. 2020). The 2016–2018 Comprehensive National Nutrition Survey (CNNS) found that 15%–40% of adolescent females and males aged 10‐19 years are deficient in one or more of the nutrients needed for hemoglobin synthesis, including iron. Notably, 31% of adolescent females were found to be iron deficient (Ministry of Health and Family Welfare MoHFW 2019).

Food fortification, by adding micronutrients to foods while food is being processed, is an intervention that can effectively reduce nutritional anemia by increasing the nutrient intake in individuals' daily diets when properly implemented (Kapur et al. 2002). By improving diet and subsequent hemoglobin concentration among WRA, anemia prevalence can be substantially reduced on a global scale (Rondini et al. 2022; Keats et al. 2019; Tam et al. 2020). In 2016, the Food Safety and Standards Authority of India (FSSAI) established food fortification standards and a logo for five foods, including salt with iron and iodine, and rice and wheat flour with iron and other micronutrients (Food Safety and Standards Authority of India 2024), which was later updated in October 2018 (FSSAI 2024). Guidelines were also issued to introduce these fortified foods under social safety net programs (SSNPs) like the Public Distribution System (PDS), Integrated Child Development Scheme (ICDS), and PM‐POSHAN (previously known as the Mid‑Day Meal/MDM program) (Duggal et al. 2022). The PDS provides subsidized food grains to low‐income households through ration shops, while ICDS delivers nutrition, health services, and preschool education to children under six and pregnant or lactating women through community centers. PM‐POSHAN provides free school meals to children in government and government‐aided schools to improve nutrition and school attendance.

Existing studies have evaluated or modeled the impact of food fortification on dietary outcome and anemia prevalence in India (Haas et al. 2014; Chakrabarti et al. 2019; Madhari et al. 2020; Swaminathan et al. 2019; Wirth et al. 2016); however, these studies are either regional studies or simulation studies based on household consumption data, requiring a series of assumptions. To date, no study has used nationally representative 24‐h dietary recall survey data to simulate the effects of fortified food in India on dietary iron intake, hemoglobin concentration, and anemia prevalence across the entire population of India, rather than focusing on a specific region or subregion.

The primary objective of the study is to model the impact of iron‐fortified rice, wheat flour, and salt on iron intake, hemoglobin concentration, and anemia prevalence in Indian WRA. We hypothesize that fortification of these foods with iron will increase iron intake, which will lead to increased hemoglobin concentration and reduced anemia prevalence among WRA in India.

## 2 Methods

We followed the STROBE (Strengthening the Reporting of Observational Studies in Epidemiology) checklist for observational and cross‐sectional studies for this paper (Checklists 2024). The methods build upon the framework developed in a thesis at Emory University (Lambert et al. 2022) and are described in detail below.

### 2.1 Settings and Data Sources

Due to the lack of a single survey containing both dietary and hemoglobin data from the same individuals, we conducted secondary analyses of two publicly available and nationally representative cross‐sectional datasets: the National Nutrition Monitoring Bureau (NNMB) from 2009 to 2012 (Global Dietary Database 2024) for food and iron intake, and the National Family Health Surveys 4 (NFHS‐4) from 2015 to 2016 for hemoglobin concentration and anemia status (International Institute for Population Sciences IIPS and ICF 2024).

The NNMB dataset, a nationally representative survey of rural areas in India, was collected using multistage stratified sampling and is publicly available for download from the Global Dietary Database (Global Dietary Database 2024). This survey utilized probability sampling without the application of survey weights. The methodology of the NNMB study has been previously detailed (Global Dietary Database 2024). Data collection included demographic information and a single, 24‐h dietary recall. Our analysis included non‐pregnant and non‐lactating women 15–49 years old with valid dietary recalls (*n* = 11,625).

In the NNMB dataset, we estimated the percentages of rice, wheat flour, and salt in each food and beverage item using personal communication from nutrition experts (Chadha et al. 2022) and then calculated the amount of rice, wheat flour, and salt consumed. This comprehensive methodology involved two key steps. First, we identified all 847 food items in the NNMB database that have rice, wheat flour, or salt. Second, we worked collaboratively with nutrition professionals who have extensive knowledge of Indian cooking practices across different regions to ensure the accuracy of ingredient proportions through expert consultation. Examples of our food decomposition methodology include chapati/roti containing 67% wheat flour, 1% salt, and 32% water; cooked rice containing 100% rice; bread containing 65% wheat flour, 2% salt, 28% water, and 5% other ingredients such as oil, sugar, and preservatives; buns containing 67% wheat flour, 1% salt, and 32% other ingredients; and rice‐based mixed dishes where rice content varied from 45% to 85% depending on preparation method. As we also estimated iron intake from both foods and beverages in WRA, in the following text, foods will be used to denote foods, beverages, and condiments.

We used data from the NFHS‐4 for hemoglobin analysis (International Institute for Population Sciences IIPS and ICF 2024). The details of the NFHS‐4 study have also been previously described (International Institute for Population Sciences IIPS and ICF 2024). NFHS‐4 is representative at both the national and state levels with data weighted by state, urban/rural area, and within major cities by slum/non‐slum status. The data are available for public download from the Demographic and Health Surveys (DHS) Program (International Institute for Population Sciences IIPS and ICF 2024). Hemoglobin was measured on capillary blood samples using HemoCue and adjusted for smoking and altitude.

### 2.2 Variables

Our outcome of interest from the NNMB data was the daily mean iron intake of WRA. From the NFHS‐4 dataset, the variables of interest were WRA's adjusted hemoglobin concentration and the prevalence of anemia, defined as hemoglobin < 120 g/L.

### 2.3 Data Analysis

We modeled the effect of fortification on iron intake, hemoglobin concentration, and anemia prevalence in non‐pregnant, non‐lactating women 15–49 years old. All analyses were conducted using SAS version 9.4 (SAS Institute Inc 2023). In the following paragraphs, we describe the methods used to model the effect of fortification on iron intake, hemoglobin concentrations, and prevalence of anemia.

### 2.4 Modeling the Effect of Fortification on Iron Intake

#### 2.4.1 Fortification Scenarios and Iron Intake Calculation

For the no‐fortification scenario, we estimated the mean iron intake for WRA from the NNMB. The intermediate and maximum scenarios were based on the fortification standards for rice, wheat flour, and salt fortification, regulated by FSSAI (FSSAI 2024). FSSAI's guidelines on fortified foods provided a range of fortification level: for fortified rice and wheat flour, we used a low fortification level of 28 mg/kg iron and a high fortification level of 42.5 mg/kg iron; for fortified salt, we used a low fortification level of 850 mg/kg iron and a high fortification level of 1100 mg/kg iron (The Food Safety and Standards Authority of India FSSAI 2024).

#### 2.4.2 Fortification Standards and Coverage Assumptions

Nutrition experts from Nutrition International estimated the reasonable percentages of the food that could be fortified by the food industry for distribution under the SSNPs and commercial market. We defined compliance as the proportion of eligible food that meets fortification standards when processed by the food industry, and coverage as the proportion of the population with access to fortified foods through both government programs and commercial distribution channels. Based on expert input, we developed an intermediate fortification scenario with realistic coverage levels: 50% coverage for rice and wheat flour and 26.6% coverage for salt (Cyriac et al. 2020), with 100% industry compliance assumed for all fortified foods (Jain and Kumar 2022). The maximum fortification scenario assumed 100% population coverage through universal implementation across both SSNPs and commercial market, and 100% industry compliance, meaning that 100% of women who consume fortifiable foods (rice, wheat flour, and salt) would have access to fortified versions, and 100% of the fortifiable food would be fortified according to government standards (Jain and Kumar 2022).

#### 2.4.3 Simulated Iron Intake Calculation Method

For the intermediate and maximum fortification scenarios, we added the amount of iron from fortified food to daily iron intake. First, we calculated the intake of iron contributed by fortification of the three staple foods (rice, wheat flour, and salt) by multiplying individual consumption amounts by the respective coverage for each scenario. Second, we multiplied this intake by the low and high fortification levels to obtain the additional iron consumed from fortifiable food. Finally, we calculated the total iron intake by summing naturally occurring iron with the additional iron from fortifiable foods. We used the FSSAI range of fortification levels to provide uncertainty bounds for our estimates, with the low and high fortification levels representing the minimum and maximum potential impacts of the intervention under each scenario described below. This range was also applied to calculate the corresponding hemoglobin changes and anemia prevalence reductions, providing uncertainty bounds for these health outcomes. Please see the mathematical representation below,

TotalIron=Fe_baseline+[(Rice ×C_rice×FL_rice/1000)+(Wheat ×C_wheat×FL_wheat/1000)+(Salt ×C_salt×FL_salt/1000)]

where:

- Fe_baseline = naturally occurring iron intake (mg/day)
- Rice, Wheat, Salt = daily intake of each food (g/day)
- C_rice, C_wheat, C_salt = coverage rates for each food by scenario
- FL_rice, FL_wheat, FL_salt = fortification levels (mg/kg)

**Scenario Parameters**

- *Intermediate*: C_rice = 0.5, C_wheat = 0.5, C_salt = 0.266. Coverage rates of 50%, 50%, and 26.6% for rice, wheat, and salt, respectively
- *Maximum*: C_rice = 1.0, C_wheat = 1.0, C_salt = 1.0. Coverage rates of 100% for all foods
- *Fortification levels*: Low (28, 28, 850 mg/kg) to High (42.5, 42.5, 1100 mg/kg) for rice, flour, and salt, respectively.

This approach enabled us to estimate iron intake ranges for each scenario, which we then used to calculate corresponding changes in hemoglobin levels and anemia prevalence reductions. We generated descriptive statistics, including the mean and standard deviation, for iron intake under both low and high fortification levels of iron in the intermediate and maximum fortification scenarios.

### 2.5 Modeling the Effect of Fortification With Iron on Hemoglobin Concentration and Anemia Prevalence

To estimate the potential hemoglobin increase based on iron intake, we used findings from one meta‐analyses based on 17 studies (Dorbu et al. 2026). Specifically, each 1 mg/day increase in iron consumed results in a mean increase in hemoglobin of 0.194 g/L (95% Credibility Intervals: [0.009,0.446]) (Dorbu et al. 2026).

For both the intermediate and maximum scenarios, we added the increased hemoglobin due to increased dietary iron intake from fortification to the hemoglobin concentration in WRA. We then calculated anemia prevalence in the no‐fortification, intermediate‐fortification, and maximum‐fortification scenarios using the WHO recommended cutoffs (hemoglobin < 120 g/L) (World Health Organization 2020).

### 2.6 Ethics Statement

We submitted the project protocol to the Emory University Institutional Review Board for consideration. Due to the de‐identified nature of the secondary datasets used in the analyses, the board determined that the research does not constitute human subjects research, and no further review was required.

## 3 Results

Women's mean age was similar in both the NNMB (Global Dietary Database 2024) and NFHS‐4 (International Institute for Population Sciences IIPS and ICF 2024), with a mean age of around 30 years (Table 1). In the NFHS‐4 dataset, 11.4% of the participants completed college, compared to only 6.7% of participants in the NNMB dataset.

**Table 1:** Select sociodemographic characteristics of the non‐pregnant, non‐lactating women 15–49 years from the NNMB Rural Survey dataset and the NFHS‐4 dataset in India^a^.

Characteristic	NNMB Rural Survey (*N* = 11,625)	NFHS‐4 2015‐2016 (*N *= 667,258)
Age (years), mean (SD)	31.2 (9.8)	30.1 (9.9)
No formal education, *n* (%)	4216 (36.4)	188,407 (28.2)
Education levels, *n* (%)		
Lower primary education (grades 1–4)	891 (7.7)	84,010 (12.6)^b^
Upper primary education (grades 5–8)	2404 (20.7)	
Secondary education (grades 9–12)	3321 (28.6)	338,853 (47.8)^b^
College and above	777 (6.7)	75,988 (11.4)^b^

^a^ NNMB, National Nutrition Monitoring Bureau (Global Dietary Database 2024); NFHS‐4, National Family Health Surveys 4 (International Institute for Population Sciences IIPS and ICF 2024).

^b^ The NFHS categorized education levels as primary school, secondary school, and education higher than secondary school, corresponding to lower and upper primary education, secondary education, and college and above in the NNMB, respectively.

In the no‐fortification scenario, women's mean (SD) daily iron intake was 12.3 mg (7.4) (Table 2). Under the intermediate scenario with 50% coverage for rice and wheat flour and 26.6% coverage for salt, with 100% industry compliance, the predicted iron intake increased to between 16.6 (8.0) and 18.8 (8.5) mg/d, representing a 35%–53% increase compared to the no‐fortification scenario. In the maximum fortification scenario, with 100% coverage and compliance, the daily iron intake could range from 20.9 (9.1) to 25.3 (10.5) mg, representing a 70%–106% increase over the no‐fortification scenario.

**Table 2:** Mean (SD) daily intake of iron intake among non‐pregnant, non‐lactating women 15–49 years in India, by fortification scenario.

	No fortification^a^ mean (SD)	Intermediate fortification^b^^,^ d mean (SD)	Maximum fortification^c^^,^ d mean (SD)
Iron intake (mg)	12.3 (7.4)	16.6 (8.0)–18.8 (8.5)	20.9 (9.1)–25.3 (10.5)
Iron added through fortification (mg/d)^d^	—	4.3 (2.1)–6.5 (3.2)	8.6 (4.2)–13.1 (6.4)
Increase (%)	—	35%–53%	70%–106%

^a^ No fortification represents the current iron intake from non‐fortified foods among women in the NNMB dataset (Global Dietary Database 2024).

^b^ Intermediate fortification assumes 50% coverage for rice and wheat flour and 26.6% coverage for salt, with 100% industry compliance for all foods. This is in addition to the iron from non‐fortified foods.

^c^ Maximum fortification represents intake after 100% population coverage and 100% industrial compliance to iron in standards. This is in addition to the iron from non‐fortified foods.

^d^ Range of the impact of fortification on iron intake based on low and high levels of iron in fortification standards issued by the Food Safety and Standards Authority of India (FSSAI): 28–42.5 mg/kg for rice and wheat flour, and 850–1100 mg/kg for salt (The Food Safety and Standards Authority of India FSSAI 2024).

Table 3 presents the predicted hemoglobin changes and anemia prevalence associated with additional iron intake from fortified foods in non‐pregnant, non‐lactating women 15–49 years in India, under both intermediate and maximum fortification scenarios. The additional iron intake from fortified food would increase hemoglobin concentration by 2.4 (1.2)–3.7 (1.8) g/L for the intermediate fortification scenario and by 4.9 (2.4)–7.4 (3.6) g/L for the maximum fortification scenario. These changes in hemoglobin concentration are predicted to have a substantial impact on anemia prevalence. In the no‐fortification scenario, anemia prevalence is 52.6%. In the intermediate fortification scenario, anemia prevalence would be reduced to 45.1%–47.6%, a 5.0–7.5 percentage points (pp) reduction compared with no fortification. In the maximum‐fortification scenario, anemia prevalence would be reduced to 35.6%–42.7%, a 10–17 pp reduction compared with the no‐fortification scenario.

**Table 3:** Hemoglobin and anemia in non‐pregnant, non‐lactating women (15–49 y) in India, by fortification scenario.

Indicator	No fortification	Intermediate fortification^a^^,^ b	Maximum fortification^a^^,^ c
Hemoglobin (g/L), mean (SD)	116.4 (16.2)	119.0 (15.8)–119.9 (15.5)	120.7 (15.2)–122.4 (14.6)
Hb increase (g/L), mean (SD)	—	2.4 (1.2)–3.7 (1.8)	4.9 (2.4)–7.4 (3.6)
Anemia, %	52.6	45.1–47.6	35.6–42.7
Anemia, *n*	350,961	301,034–317,407	237,657–284,652
Percentage point decrease, %	—	5.0–7.5	10.0–17.0

^a^ Range of the impact of fortification on iron intake based on low and high levels of iron in fortification standards issued by the Food Safety and Standards Authority of India (FSSAI): 28‐42.5 mg/kg for rice and wheat flour, and 850‐1100 mg/kg for salt.

^b^ Intermediate fortification assumes 50% coverage for rice and wheat flour and 26.6% coverage for salt, with 100% industry compliance for all foods. This is in addition to the iron from non‐fortified foods.

^c^ Maximum fortification represents intake after 100% population coverage and 100% industrial compliance to iron in standards. This is in addition to the iron from non‐fortified food.

## 4 Discussion

This study modeled the potential impact of the combined fortification of rice, wheat flour, and salt, based on the India government standards, on iron intake and hemoglobin concentration among non‐pregnant, non‐lactating WRA (15–49 y), compared with no fortification. We estimated how additional iron from fortified foods would increase hemoglobin concentration and decrease anemia prevalence among WRA under two fortification scenarios: an intermediate scenario with 50% program coverage for rice and wheat flour and 26.6% for salt and 100% compliance for the three fortification vehicles, and a maximum scenario with 100% coverage and compliance of the three vehicles. Our results suggest a 35%–53% increase in iron intake in the intermediate scenario and a 70%–106% increase in the maximum scenario. Anemia prevalence is projected to decrease by 5.0–7.5 pp in the intermediate and 10–17 pp in the maximum scenarios due to the combined fortification.

These results support our initial hypothesis: consumption of rice, wheat flour, and salt fortified with iron, as per the Government of India standards, is expected to increase iron intake and hemoglobin concentration in women, thereby reducing the overall prevalence of anemia. India's national program aims to reduce anemia by 3 percentage points (pp) annually (Thankachan 2024), which aligns with our findings, where the intermediate scenario predicts a reduction in anemia prevalence by 5.0–7.5 pp, while the maximum intervention scenario predicts a reduction of 10–17 pp.

In comparison to previous literature, we identified two other simulation studies assessing the potential impact of iron‐fortified foods on iron intake among WRA in India. Our estimates suggest more substantial reductions in anemia prevalence compared to these previous studies. Our models predict anemia prevalence decreases from baseline to 45.1%–47.6% for the intermediate scenario and 35.6%–42.7% for the maximum fortification scenario. In contrast, when we applied our fortification levels to Swaminathan et al.'s model, their approach would predict much more modest reductions—from 52.6% to 50.6%–51.1% for the intermediate scenario and to 49.3%–50.3% for the maximum scenario (Swaminathan et al. 2019).

The difference in their simulated results compared to ours can be attributed to the different data source (household consumption surveys in both urban and rural areas in theirs and our 24‐h recalls in rural places) and different modeling approaches used: we utilized meta‐analyses (Dorbu et al. 2026; Van Cor et al. 2022) that derived the effect of iron fortification on anemia from multiple countries and assumed the hemoglobin distribution parallelly shifted, while they conducted a regression analysis of anemia on iron intake, assuming a linear relationship between the logarithm of anemia odds and iron intake.

Wirth conducted a cross‐sectional survey in Telangana, India, and discovered that 99% of salt was iodized, and approximately 25% of rice was produced by households rather than purchased from a market or shop. This finding implies that rice fortification can reach 75% of the rural households in this state and potentially higher in states that do not produce rice, even though fortified rice could contribute > 80% of Recommended Dietary Allowance (RDA) for many micronutrients, such as iron, zinc, thiamin, riboflavin, niacin, and vitamin A (Wirth et al. 2016). Since they also used household‐level rice purchase rather than individual rice consumption, their modeling could overestimate or underestimate the actual amount of rice consumed, and thus the effect of fortified rice on diet adequacy.

Swaminathan's and Wirth's studies, along with ours, all showed that fortification of staples and salt can improve iron intake, increase hemoglobin concentration, and reduce anemia. However, the degree of improvement varies depending on the model assumptions. While simulation models can provide valuable insights and predictions, they inherently rely on assumptions and existing data that may not fully capture real‐world complexities and variations. Longitudinal studies would provide empirical evidence on how fortified foods impact iron status and anemia rates in different settings and among diverse groups, thereby validating the predictions made by these models.

Our study had several other limitations. One primary limitation involves the estimation of food intake, which was based on the NNMB (National Nutrition Monitoring Bureau) data (Global Dietary Database 2024). This dataset represents the dietary patterns of the rural population, which might not be reflective of the urban population's dietary habits. However, Swaminathan et al. compared dietary intake data from the Household Consumer Expenditure Survey (both urban and rural household consumption) to dietary intake from rural households in the NNMB and found a strong correlation (*r* = 0.81, *p* < 0.001) between surveys (Swaminathan et al. 2019). This suggests that rural intakes are comparable to both rural and urban intakes, supporting the relevance of our findings across different populations. An important limitation of our study involves the use of single‐day 24‐h dietary recalls from the NNMB dataset. While single‐day recalls are appropriate for estimating population mean iron intake—which is the primary focus of our fortification scenarios—they do not account for intra‐individual day‐to‐day variation in dietary intake. Without multiple days of dietary data or assumptions about intra‐person variance, we cannot make strong inferences about the distribution of usual individual intakes or identify populations at risk of inadequate or excessive intake. The standard deviations we report in our results reflect inter‐individual variation but incorporate measurement error from day‐to‐day variation from the same individual, which may overestimate the true variability in usual intakes. Therefore, our estimates of mean iron intake changes under different fortification scenarios should be interpreted at the population level, while acknowledging that individual‐level intake distributions and the proportion of the population at risk of inadequate or excess cannot be precisely estimated from these data.

Additionally, while simulation models can provide valuable insights and predictions, they inherently rely on assumptions and existing data that may not fully capture real‐world complexities and variations. Our modeling approach assumed a linear relationship between iron intake and hemoglobin concentration based on meta‐analytical evidence, though the actual biological relationship may involve some non‐linearity, particularly at higher iron intake levels or in populations with varying iron status. Furthermore, our analysis provides national‐level estimates and does not account for state‐specific variations in dietary patterns, anemia prevalence, or program implementation capacity, which may limit the applicability of our findings for state‐level policy planning and resource allocation.

While our modeling demonstrates substantial potential for anemia reduction, successful implementation requires attention to several factors. A small proportion of individuals with genetic conditions (e.g., thalassemia) may be sensitive to increased iron intake (World Health Organization 2006). However, existing fortification programs in multiple countries using moderate fortification levels similar to FSSAI standards have not shown population‐level iron overload. Monitoring iron biomarkers can identify any unexpected effects. Iron fortification operates alongside other sources, including IFA supplementation, naturally iron‐rich diets, and environmental iron. FSSAI fortification levels were designed considering total dietary patterns in India, and coordinating interventions through dietary surveillance can prevent excessive exposure (The Food Safety and Standards Authority of India FSSAI 2024).

Regarding children who consume fortified staples, evidence on safety in malaria‐endemic areas is important to consider. While early studies raised concerns about infection risks from high‐dose iron supplementation, a systematic review of six home fortification studies (which provides higher iron intake than food fortification) in malaria‐endemic areas found no increased risk of morbidity, including malaria (Dewey and Baldiviez 2012). In summary, given the substantial anemia burden in India and demonstrated global effectiveness, iron fortification of rice, wheat flour, and salt represents a valuable public health strategy. Implementation with routine monitoring using existing infrastructure represents standard responsible practice and can achieve the projected anemia reductions.

Our study has several strengths, particularly in the context of the recent May 2023 WHO/World Health Assembly resolution on food fortification (New WHA resolution to accelerate efforts 2024). This resolution underscored the urgency of accelerating large‐scale fortification to combat micronutrient deficiencies, a goal to which our study directly contributes. The modeling exercise conducted in our study plays a pivotal role in understanding the potential impacts of food fortification. Without such predictive models, the practical effects of food fortification efforts on public health cannot be accurately anticipated and strategized. Additionally, our study used nationally representative 24‐h recall data, instead of sub‐national or regional surveys or household food consumption surveys. The use of individual 24‐h dietary recall data provides substantial methodological advantages over household consumption surveys for modeling food fortification impacts. Unlike household consumption studies that rely on adult male equivalent conversions and assume uniform intra‐household food distribution (Gibson and Ferguson 2008; Smith et al. 2006), our approach captures actual consumption patterns among WRA without requiring distributional assumptions. Previous studies using household expenditure data have acknowledged limitations in accurately estimating individual‐level intake for vulnerable groups such as women and children who may have differential access to household food resources (Adams et al. 2022), which is particularly important for fortification modeling given that consumption patterns can vary significantly within households based on age, gender, and physiological status (Kennedy and Peters 1992).

In addition, we modeled the effect of both intermediate and maximum fortification scenarios which add a layer of depth to the analysis. By providing these two distinct scenarios and using national surveys, we offer a comprehensive view of the potential outcomes of fortification efforts. The intermediate scenario presents a realistic picture of what could be achieved with partial coverage and full compliance, while the maximum scenario illustrates the best‐case outcomes with full implementation. By understanding the range of possible outcomes, the stakeholders can better strategize, allocate resources, and set realistic targets for their fortification initiatives.

Our study suggests that combined fortification of rice, wheat flour, and salt with iron under the maximum scenario can be an effective method of decreasing nutritional anemia among WRA in India. Even with the intermediate scenario, which may be more realistic for coverage, we can still expect a reduction of anemia from 52.6% among women to between 45.1% and 47.6% if these staples are mass fortified in India.

## Author Contributions

H.L. and E.L. conducted the research. H.P. and M.C. designed the study. H.L. and X.L. performed data analysis and interpretation. E.L. prepared the initial draft of the manuscript with support from H.P. and M.C. H.L. re‐analyzed the data, extensively revised, and rewrote the manuscript. All authors contributed to the manuscript revision and approved the final version for publication.

## Conflicts of Interest

The authors declare no conflicts of interest.

## Data Availability

The authors have nothing to report.
